# Peroxisome proliferator‐activated receptor gamma‐ligand‐binding domain mutations associated with familial partial lipodystrophy type 3 disrupt human trophoblast fusion and fibroblast migration

**DOI:** 10.1111/jcmm.15401

**Published:** 2020-06-09

**Authors:** Hussein Shoaito, Sabine Chauveau, Camille Gosseaume, William Bourguet, Corinne Vigouroux, Camille Vatier, Catherine Pienkowski, Thierry Fournier, Séverine A. Degrelle

**Affiliations:** ^1^ INSERM UMR‐S1139 (3PHM) Université de Paris Paris France; ^2^ Laboratoire ICARE Biopôle Clermont‐Limagne Saint‐Beauzire France; ^3^ Inserm UMR‐S938 Department of Endocrinology, Diabetology and Reproductive Endocrinology Saint‐Antoine Research Centre Institute of Cardiometabolism and Nutrition (ICAN) AP‐HP Saint‐Antoine Hospital National Reference Centre of Rare Diseases of Insulin Secretion and Insulin Sensitivity (PRISIS) Sorbonne Université Paris France; ^4^ INSERM CNRS Centre de Biochimie Structurale (CBS) Université de Montpellier Montpellier France; ^5^ Department of Molecular Biology and Genetics AP‐HP, Saint‐Antoine Hospital Paris France; ^6^ Endocrinology Unit Reference Centre for Rare Gynecologic Diseases Toulouse France; ^7^ PremUp Foundation Paris France; ^8^ Inovarion Paris France

**Keywords:** cell fusion, cell migration, familial partial lipodystrophy type 3 (FPLD3), Placenta, PPARG, trophoblast differentiation

## Abstract

The transcription factor peroxisome proliferator‐activated receptor gamma (PPARG) is essential for placental development, and alterations in its expression and/or activity are associated with human placental pathologies such as pre‐eclampsia or IUGR. However, the molecular regulation of PPARG in cytotrophoblast differentiation and in the underlying mesenchyme remains poorly understood. Our main goal was to study the impact of mutations in the ligand‐binding domain (LBD) of the *PPARG* gene on cytotrophoblast fusion (PPARG^E352Q^) and on fibroblast cell migration (PPARG^R262G^/PPARG^L319X^). Our results showed that, compared to cells with reconstituted PPARG^WT^, transfection with PPARG^E352Q^ led to significantly lower PPARG activity and lower restoration of trophoblast fusion. Likewise, compared to PPARG^WT^ fibroblasts, PPARG^R262G^/PPARG^L319X^ fibroblasts demonstrated significantly inhibited cell migration. In conclusion, we report that single missense or nonsense mutations in the LBD of PPARG significantly inhibit cell fusion and migration processes.

## 1 INTRODUCTION

Peroxisome proliferator‐activated receptor gamma (PPARG) is a nuclear receptor involved in lipid metabolism,^1^, ^2^ insulin resistance^3^ and inflammation.^3^ Furthermore, experiments with knockout mice have demonstrated the importance of PPARG for placental formation and gestational outcome.^4^, ^5^ In humans, the placenta is composed of a mesenchymal core covered by mononucleated villous cytotrophoblasts (VCTs) attached to the villous basement membrane. These cells fuse and renew throughout pregnancy the multinucleated syncytiotrophoblast (ST) layer. The ST, which forms the outermost surface of the placental chorionic villi, is in direct contact with maternal blood within the intervillous space. The epithelial layer of VCT and the outer layer ST constitute the placental barrier at the interface between maternal and fetal circulation. This multinucleated layer regulates gas and nutrient exchange, supports intensive endocrine functions and provides immunological support to the fetus. In the human placenta, PPARG is highly expressed in trophoblasts and is directly involved in the differentiation of both villous and extravillous cytotrophoblasts.^6^, ^7^ Thus, abnormal PPARG expression and/or activity is likely to result in placental dysfunction and pregnancy complications such as pre‐eclampsia and intra‐uterine growth retardation.^8^, ^9^, ^10^

PPARG comprises a DNA‐binding domain, an agonist‐independent activation domain (AF‐1) and an agonist‐dependent activation domain (AF‐2), which contains the ligand‐binding domain (LBD). PPARs heterodimerize with the retinoid X receptor (RXR)‐α and activate the transcription of target genes by binding to the PPAR response element (PPRE). The transcriptional activity of PPARG is principally modulated by agonists, which recruit either coactivators or corepressors. In general, ligand‐bound PPARG recruits coactivators, whereas ligand‐free PPARG is bound to corepressors.

**Figure 1:**
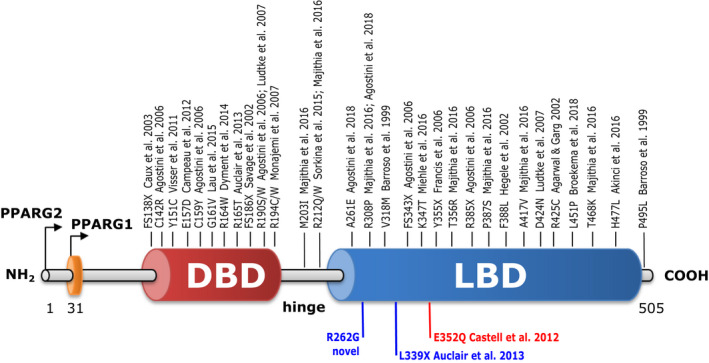
Genomic maps of distinct domains of PPARG highlighting mutations reported from patients with familial partial lipodystrophy type 3 (FPLD3). In red, the E352Q mutation associated with villous cytotrophoblast fusion. In blue, the R262G and L339X mutations implicated in skin fibroblast migration

Six types of familial partial lipodystrophy (FPLD) have been recorded in the literature.^11^, ^12^, ^13^, ^14^, ^15^ Of these, FPLD type 3 (FPLD3) is caused by rare autosomal dominant mutations in the PPARG gene.^16^ To date, 35 mutations have been linked to FPLD3 ^17^ (Figure 1). FPLD3 patients are characterized by aberrant adipose tissue distribution and severe metabolic complications, including diabetes.^11^ FPLD3‐causing mutations can have multiple effects on metabolic phenotypes and numerous downstream complications. It is no coincidence that pharmacological agonists of PPARG have been shown to improve insulin resistance, reduce the intermediate metabolic disturbances and likely reduce cardiovascular end‐points.^18^

In this study, we were interested in cell fusion and migration, two processes that are essential for placental development. First, we examined a mutation in PPARG, PPARG^E352Q^, which is known to be associated with FPLD3. This mutation was first described in a report of placental abnormalities that led to premature delivery of a baby who developed hydrops foetalis and died 24 hour after delivery. The infant's karyotype was normal (46 XX) but placental DNA analysis revealed the presence of the same heterozygous mutation in the PPARG‐LBD as in the mother.^19^ Here, we specifically investigated by a combination of knockdown and reconstitution approach, the effect of this mutation on in vitro differentiation of villous cytotrophoblast, *that is* formation of ST by a cell‐cell fusion process. We also evaluated the impact on the migration of mesenchymal cell (fibroblast) isolated from two patients carrying mutations in the LBD of PPARG, the novel missense mutation PPARG^R262G^ and the previously described nonsense mutation PPARG^L339X^, which has been linked with a defect in transrepression of cellular RAS and consequent cellular dysfunction.^20^

## 2 MATERIALS AND METHODS

### 2.1 Ethical statement

The study was performed according to the principles of the Declaration of Helsinki. Placentas were obtained with the patients’ written informed consent, and the protocol was approved by the local ethics committee (CPP 2015‐mai‐13909). Placental tissues were obtained from women with uncomplicated pregnancies who underwent scheduled caesarean section at the Cochin Port‐Royal, Antony and Montsouris maternity units (Paris, France). Wild‐type and PPARG‐mutated skin fibroblasts were obtained from the PRISIS collection (http://endocrino‐sat.aphp.fr/prisis/). Briefly, primary skin fibroblast cultures had been established following superficial punch biopsy of two groups of patients: (a) two FPLD3 patients who harboured PPARG mutations (R262G or L339X) and, (b) as wild‐type controls, three non‐obese, non‐diabetic, normotensive women without any cardiovascular disease who were undergoing plastic surgery. All subjects gave their written informed consent for these studies, which were approved by an institutional review committee.

### 2.2 siRNA and mammalian expression vectors

siRNA transfections were performed as described in^21^ with stealth PPARG siRNA (VHS40941 or VHS40944, Life Technologies). Of the two stealth siRNAs, VHS40944 was selected as the best (sense PPARG5′‐GCUUCAUGACAAGGGAGUUUCUAAA‐3′ and antisense 5′‐CGAAGUACUGUUCCCUCAAAGAUUU‐3′). A negative scrambled siRNA (stealth RNAi siRNA negative control med GC, Life Technologies, France) was used in parallel as control. Knockdown efficiency was determined by immunoblotting for siRNA that targeted the PPARG protein.

The expression plasmid for PPARG^E352Q^ was generated by directed mutagenesis of the pcDNA3‐PPARG^WT^ plasmid. The PPARG^WT^ and PPARG^E352Q^ inserts were amplified by PCR using SalI‐PPARG_F 5′‐AT(GTCGAC)ACCATGGTTGACACA‐3′ and KpnI‐PPARG_R 5′‐CGG(GGTACC)CTAGTACAAGTCCTTGTAGATC‐3′, and then cloned into the pEGFP‐C1 vector using the restriction sites SalI and KpnI. The pEGFP‐C1 vector was kindly provided by Jean‐Baptiste Brault (Institut Curie). PPARG siRNA insensitive clones (pEGFP‐PPARG*^WT^ and pEGFP‐PPARG*^E352Q^) were generated by introducing three nucleotide switches: C1038T, A1048C and A1059C. All constructs were verified by sequencing. For all expression vectors, transfection efficiency was determined to be ~45% for VCT and ~60% for NIH/3T3 cells.

The secreted mCherry reporter, CMV‐ss.mCherry plasmid, was constructed based on.^22^ Briefly, the N‐terminal signal sequence of a GPI‐anchored T‐cadherin was fused to the N‐terminus of mCherry. The ss.mCherry insert was amplified by PCR using KpnI‐ss.mCherry_F 5′‐CGG(GGTACC)ATGCAGCCGAGAACTCCGCTCACCCTGTGCGTCCTGCTGTCCCAGGTGCTCCTGGTAACATCTGCAGTGAGCAAGGGCGAGGAG‐3' and Notl‐m Cherry%R5. The fusion ss.mCherry protein was used as a transfection control for the nanoluciferase assay, when cells were transfected, ss.mCherry was secreted into the cell culture media and quantified in tandem with luminescence by an EnSpire Multimode plate reader (Perkin Elmer).

### 2.3 Cell culture, transfection and treatment

NIH/3T3 cells (ATCC #CRL‐1658) were cultured in complete DMEM (1% glutamine, 1% penicillin‐streptomycin and 10% foetal calf serum) in 5% CO_2_ at 37°C. After 24 hour incubation, cells were washed and incubated for 2 hour with 1 µmol/L GW1929 that was diluted in culture medium containing 1% glutamine and 10% foetal calf serum (without penicillin‐streptomycin). Then, NIH/3T3 cells were transfected with [eGFP‐]PPARG^WT^, [eGFP‐]PPARG^E352Q^, pSG5‐RXRα, and reporters PPRE‐Histone2B(H2B)‐eGFP (Addgene #84393)/pDEST‐lifeact‐mCherry (Addgene #40908^23^) or PPRE‐pNL1.3[secNluc] (Addgene #84394)/CMV‐ss.mCherry, using Lipofectamine 3000 (Invitrogen) and following the manufacturer's protocol. Six hours later, the transfected NIH/3T3 cells were washed and treated with 1 µmol/L GW1929 diluted in culture medium containing 1% glutamine, 10% foetal calf serum and 1% penicillin‐streptomycin for 48 hours. VCTs were isolated from human term placentas (n = 7) by sequential enzyme digestions and a Percoll gradient as previously described in.^21^, ^24^, ^25^ VCTs were cultured in DMEM (1% glutamine, 1% penicillin‐streptomycin and 10% foetal calf serum) in 5% CO_2_ at 37°C. After overnight incubation, cells were washed, incubated 2 hours with Opti‐MEM I medium without serum and then transiently transfected with either the eGFP‐PPARG^WT^ or eGFP‐PPARG^E352Q^ plasmid along with PPARG siRNA, using Lipofectamine 2000 (Invitrogen) and following the manufacturer's protocol. After 6 hours, transfected VCTs were washed and cultured with complete DMEM (1% glutamine, 1% penicillin‐streptomycin and 10% foetal calf serum) for 48 hours. After 72 hours of culture, NIH/3T3 cells and VCTs were preserved in one of two ways: (a) 4% paraformaldehyde‐fixed, washed and stored in 1× phosphate buffered saline (PBS) at 4°C until immunostaining was performed, or (b) snap‐frozen, then stored at −80°C until Western blot analyses were conducted.

### 2.4 Nanoluciferase assay

NIH/3T3 transfected with eGFP‐PPARG^WT^ or eGFP‐PPARG^E352Q^, pSG5‐RXRα, and reporters pNL1.3[secNluc]/CMV‐ss.mCherry, were cultured in triplicate in a 24‐well plate. After 48 hours of GW1929 treatment, 100 μL of each cell supernatant was dispensed into the wells of a 96‐well black plate (#3631, Corning). The amount of secreted NanoLuc^®^ luciferase activity was determined using the Nano‐Glo^®^ Luciferase Assay (#N1130, Promega Corporation) based on the manufacturer's instructions. Luminescence and fluorescence of secreted mCherry (Ex:587nm, Em:610nm) were measured in each well using an EnSpire Multimode plate reader (Perkin Elmer). Each luminescence reading was normalized to the corresponding mCherry signal.

### 2.5 Migration assay

Wild‐type (n = 3) and PPARG‐mutated (n = 2, PPARG^R262G^, PPARG^L339X^) skin fibroblasts were cultured in DMEM/F12 (1% glutamine, 1% penicillin‐streptomycin and 10% foetal calf serum). For the migration assay, cells were seeded at 5 × 10^4^ cells/well in a fibronectin‐coated 96‐well ImageLock Plate (Essen Bioscience). After overnight incubation, cells were 95%‐100% confluent, and scratch wounds (~200 µm) were made using the accompanying WoundMaker™ device (Essen Bioscience). Plates were immediately placed into an automated live‐cell imager (IncuCyte Zoom; Essen Bioscience). Images were recorded every 30 minutes for 24 hours. Experiments were conducted at 37°C with 5% CO_2_. IncuCyte Zoom software was used to analyse the images and determine the rate of cell migration, graphed as the percentage of Relative Wound Density (RWD), whereas area under the curve (AUC) was calculated for each condition from mean RWD values to compare the fibroblasts.

### 2.6 Immunofluorescence and image analysis

After 72 hours of culture, cells cultured on 8‐well removable chamber (#80841/Ibidi) were fixed in 4% paraformaldehyde for 20 minutes at room temperature, washed in 1× PBS and permeabilized in 0.5% Triton X‐100 in PBS for 30 minutes. Then, cells were blocked in 5% bovine serum albumin (BSA‐IgG free) and 0.1% Tween‐20 in PBS for 1 hour at room temperature. For the fusion index assay, VCTs were immunostained with either Desmoplakin (DSP; ab71690, Abcam) or GATA3 (sc‐268, Santa Cruz) primary antibody as described in.^26^ For the GFP‐PPARG^WT^‐ and GFP‐PPARG^E352Q^‐transfected NIH/3T3 cells, only Alexa Fluor^®^ 555 Phalloidin and DAPI labelling were performed as described in.^27^ For analyses of cytoskeletal architecture, wild‐type and PPARG‐mutated fibroblasts were immunostained with anti‐vimentin (sc‐7557, Santa Cruz) and anti‐vinculin (V9131, Sigma) antibodies, overnight in blocking solution. The next day, all three groups of cells were rinsed three times with 0.1% Tween‐20 in PBS (PBST), and then, staining was revealed with an appropriate secondary antibody or phalloidin coupled with Alexa Fluor 488, 555 or 647 (Invitrogen) for 1 hour, in the dark at room temperature. After three washes in PBST, cells were counterstained with DAPI for 10 minutes at room temperature. Finally, slides were mounted with Fluoromount‐G (Molecular Probes) and stored at 4°C. Confocal microscopy images were obtained with a Leica SP8 inverted microscope equipped with a Plan Apo oil‐immersion ×40 objective (numerical aperture 1.30). The fusion index was calculated in three independent experiments as [100 − %number of GATA3+ nuclei/total number of DAPI nuclei], using ImageJ (NIH), as described in.^26^ Quantification of PPRE‐H2B‐eGFP was performed as described in.^27^ Briefly, a minimum of 100 nuclei were analysed per condition in three independent experiments. For quantification, fluorescent signals were integrated over the entire nucleus.

### 2.7 Western blotting

After 72 hours of culture, total cell extracts were lysed in Laemmli Buffer. Protein samples were resolved by SDS‐PAGE and immunoblotted with antibodies: anti‐PPARG (sc‐7196, Santa Cruz), anti‐GFP (AB0020, Sicgen), anti‐vinculin (V9131, Sigma‐Aldrich) and/or anti‐actin (A5441, Sigma‐Aldrich). After 1 hour incubation with the appropriate Alexa Fluor‐conjugated secondary antibody (680 or 800 conjugate, Molecular Probes), blots were revealed using the Odyssey infrared fluorescent system (Li‐Cor). Signal intensity was quantified using Image J. The arbitrary pixel densities of each protein were normalized to actin.

### 2.8 Structural analysis

Visual inspection of the PPARG structure (PDB code 5YCP) was done using COOT^28^ and Figures were prepared with PyMOL (http://pymol.org/).

### 2.9 Statistical analyses

All measurements were performed at least in three independent experiments. The data are expressed as the mean + SEM for nanoluciferase and migration assays, or mean + SD of the indicated number for Western blots and assays of the fusion index and PPRE‐H2B‐eGFP. Statistical comparison (paired *t* test) of each treated group versus control was performed using GraphPad Prism 6 software. Results were considered significant if the *P*‐value was <.05 (*), <.01 (**), <.001 (***) or <.0001(****).

## 3 RESULTS

### 3.1 E352Q mutation decreases PPARG activity

**Figure 2:**
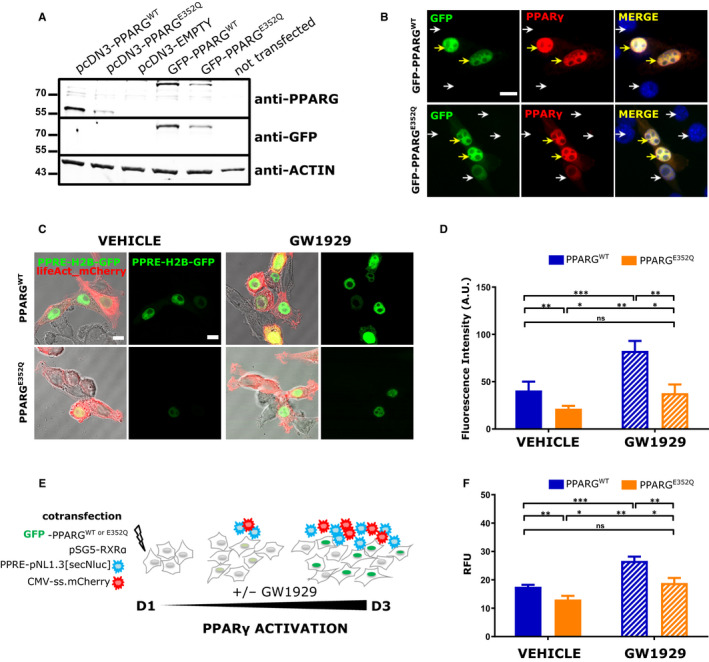
E352Q mutation reduces PPARG activity. For validation of PPARG^WT/E352Q^ expression (A, B), NIH/3T3 cells were transfected with all PPARG plasmids [pcDNA3‐PPARG^WT^, pcDNA3‐PPARG^E352Q^, GFP‐PPARG^WT^ and GFP‐PPARG^E352Q^]. After 48 h of culture, cells were subjected to A, immunoblot analysis with the indicated antibodies and B, PPARG immunostaining for GFP‐PPARG^WT^ or ^E352Q^ (Green), PPARG (Red) and nuclei (DAPI, blue). Yellow and white arrows indicate GFP+ and GFP− nuclei, respectively. Scale Bar: 10 µm. For PPARG activity assays (C‐F), NIH/3T3 cells were co‐transfected with either C‐D, PPARG^WT^ or ^E352Q^, pSG5‐RXRα, PPRE‐H2B‐eGFP (green) and lifeAct‐mCherry (red) reporter plasmids or E‐F, GFP‐PPARG^WT^ or ^E352Q^, pSG5‐RXRα, PPRE‐pNL1.3[secNluc] and CMV‐ss.mCherry reporter plasmids. C, Representative images of co‐transfected NIH/3T3 and GW1929‐treated cells. Scale Bar: 10 µm. D, Quantification of PPRE‐H2B‐eGFP fluorescence intensity. Values are presented as mean + SD of the fluorescence intensity (n = 3, >100 nuclei). E, Schema of the co‐transfection of secreted reporters (secNluc and ss.mCherry). F, PPARG activity was assayed using the Nano‐Glo^®^ Luciferase system (Promega). For each condition (triplicate), 100 µL of culture media was analysed after 48 h of treatment and luminescence signal was normalized with the corresponding mCherry signal. Values are represented as mean + SEM (n = 3 in triplicate). Statistical analysis was performed using paired *t* test to compare to vehicle control; *P < .05, **P < .01, **P < .001, ****P < .0001

To evaluate the effect of the E352Q mutation on PPARG activity, we used the NIH/3T3 cell line, which does not express PPARG. We first validated by Western blots (Figure 2A) and immunofluorescence (Figure 2B) the expression of all our PPARG^WT^ and PPARG^E352Q^ plasmids in transiently transfected cells. As expected, we observed in the Western blots, a single ~55 kD corresponding to either _(pcDNA3)_PPARG^WT^ or ^E352Q^, and a single ~89 kD band corresponding to either GFP‐PPARG^WT^ or ^E352Q^, and no endogenous PPARG (~55 kD) in _(pcDNA3)_EMPTY and not transfected controls (Figure 2A). In the immunofluorescence images, a co‐localization of GFP‐PPARG^WT^ or ^E352Q^ with the anti‐PPARG antibody in the nuclei of the transfected cells is observed (yellow arrows; Figure 2B). For the PPARG activity assays, we next cotransfected cells with PPARG^WT^ or ^E352Q^ plasmids along with reporter plasmids we developed: PPRE‐H2B‐eGFP, lifeAct‐mCherry (Figure 2C‐D) or PPRE‐pNL1.3[secNluc], CMV‐ss.mCherry (Figure 2E‐F) ^27^; PPARG cells were then treated with a PPARG agonist (1 µmol/L GW1929). In the first assay, cotransfected NIH/3T3 (lifeAct‐mCherry+) cells presented eGFP+ nuclei that reflected the intensity of PPARG transcriptional activity (Figure 2C). Compared to PPARG^WT^, PPARG^E352Q^ showed significantly lower PPARG activity in both untreated cells and cells treated with the GW1929 PPARG agonist (Figure 2D, 40.7 vs 21.5; *P* < .001; 82.6 vs 37.8; *P* < .001, respectively). This was confirmed by cotransfection of the NIH/3T3 cells with the PPRE‐pNL1.3[secNluc] plasmid (Figure 2E): there was significantly lower PPARG activity in the mutant cells regardless of treatment with PPARG agonist (Figure 2F, no GW1929: 17.5 vs 13, *P* < .001; with GW1929: 26.7 vs 18.9, *P* < .001, respectively).

### 3.2 PPARG^E352Q^ mutation decreases villous cytotrophoblast fusion

**Figure 3:**
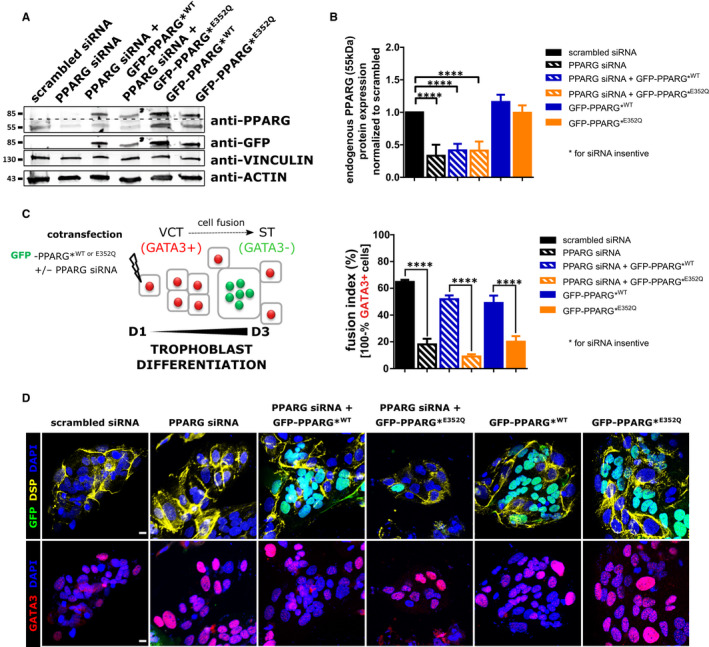
E352Q mutation reduces VCT fusion. After 16 h of culture, VCTs were co‐transfected with PPARG siRNA along with a siRNA‐resistant GFP‐PPARG^WT^ or GFP‐PPARG^E352Q^ plasmid for 6 h. After 72 h of culture, cells were subjected to A, immunoblot analysis with the indicated antibodies. Dotted lines indicate parts combined from a single gel and exposure. B, Quantification of the protein level by densitometry. Values are represented as mean + SD (n = 3). C, Fusion assay with immunostaining with anti‐GATA3 (nuclear marker of mononucleated VCT) and anti‐DESMOPLAKIN (DSP, marker of plasma membrane). Nuclei were counted manually using the 'cell counter' plugin of ImageJ. Fusion index (%) was calculated as 100 − % (number of GATA3+ nuclei/total number of nuclei). Values are represented as mean + SD of the percentage (n = 3). D, Representative images of transfected cells immunostained as in C: GFP+, (green), GATA3 (red), DESMOPLAKIN (yellow) and DAPI (blue). Scale Bar: 10 µm. Statistical analysis was performed with paired t test to compare scrambled siRNA or wild‐type control. ∗∗∗∗*P* < .0001

To investigate the effect of PPARG^E352Q^ on the in vitro differentiation of human cytotrophoblast into STs, we performed cell fusion assays on trophoblasts that were depleted of endogenous PPARG by siRNA transfection. To rescue PPARG knockdown, mammalian expression vectors were introduced that encoded PPARG^WT^ or PPARG^E352Q^ fused to GFP and that were insensitive to functional siRNA (GFP‐PPARG*^WT,^ GFP‐PPARG*^E352Q^; Figure 3). After normalization to actin or vinculin levels, knockdown of PPARG through RNA interference reduced protein expression by approximately 65% compared to cells transfected with scrambled siRNA (Figure 3A‐B; *P* < .001). As evident from the fusion assay based on GATA3 immunostaining (Figure 3C), and confocal microscopy images (Figure 3D), PPARG knockdown decreased trophoblast fusion compared to transfection with the scrambled control (18.1% vs 64.7%; *P* < .001). Furthermore, when these PPARG‐knockdown cytotrophoblasts were reconstituted with GFP‐PPARG*^E352Q^, they showed a significantly lower level of syncytialization (9% vs 51.9%; *P* < .0001) compared to those reconstituted with GFP‐PPARG*^WT^. Similar results were obtained from cytotrophoblasts that overexpressed GFP‐PPARG*^E352Q^ compared to those that overexpressed PPARG*^WT^ (20.1% vs 49.1%; *P* < .0001).

### 3.3 PPARG^R262G^ or PPARG^L339X^ mutation decreases fibroblast migration

**Figure 4:**
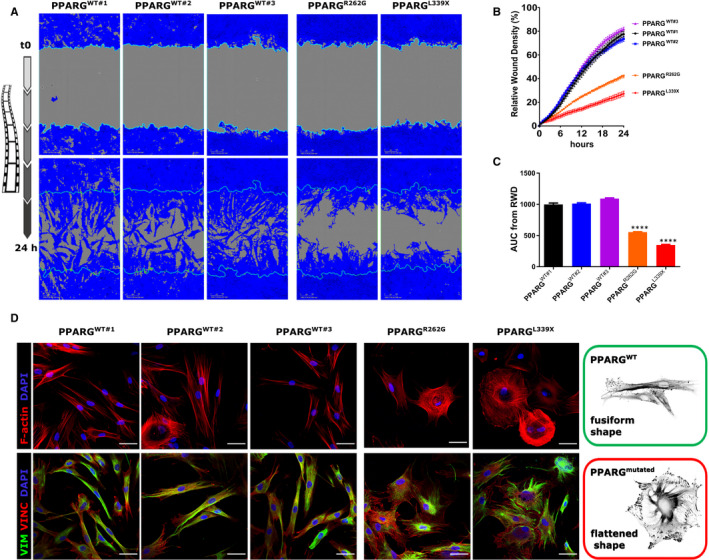
R262G and L339X mutations induce morphological changes that reduce fibroblast migration. Wild‐type and PPARG‐mutated fibroblasts were seeded at equal densities into a 96‐well plate (n = 3‐5 wells/condition), cultured to confluency, mechanically wounded by scratching and then monitored for 24 h using IncuCyte Zoom. A, Representative images of fibroblast migration at 24 h. The blue region denotes the area of the initial wound (light blue line) covered by advancing cells. B, Time course of wound closure expressed as RWD (%). Values are represented as mean ± SEM of the percentage. C, Comparison between wild‐type and PPARG‐mutated fibroblasts is shown by AUC analysis of the replicates. Values are represented as mean + SEM. Statistical analysis was performed with paired *t* test to compare wild‐type controls. ****P < .0001. D, Representative images showing immunofluorescent staining for F‐actin (red) in upper panels, and vimentin (VIM, green) and vinculin (VINC, red) in lower panels. Cell nuclei were also stained with DAPI (blue). Scale bar: 50 µm. Insets depict a cytoskeleton architecture model of wild‐type (WT, Green) and PPARG‐mutated (red) fibroblasts

We next investigated the effect of two other PPARG‐LBD mutations on the cell migration process. We performed wound‐healing assays with skin fibroblasts obtained from three control patients (PPARG^WT^) and two patients with FPLD3 (PPARG^R262G^ or PPARG^L319X^ mutations; Figure 4A‐C). Compared to the PPARG^WT^ control fibroblasts, the R262G and L339X mutations of PPARG demonstrated significantly inhibited cell migration (77.6% ± 4.1% vs 34.5% ± 10.3%; *P* < .0001). Furthermore, inhibition of PPARG^L339X^‐mutated cells was higher than those with the PPARG^R262G^ mutation (27.2 ± 5.19 vs 41.9 ± 4.3; *P* < .0001). To follow up on this observation, we examined the morphology of wild‐type and PPARG‐mutated fibroblasts. PPARG^WT^ fibroblasts had an elongated polarized form (also called fusiform shape) with numerous straight actin bundles, whereas PPARG^R262G^ and PPARG^L319X^ fibroblasts spread into flattened shapes (Figure 4D, upper panel). Interestingly, compared to PPARG^WT^, PPARG‐mutated fibroblasts possessed an edge of dot‐like focal complexes around the entire periphery of the cell (Figure 4D, lower panel).

## 4 DISCUSSION

**Figure 5:**
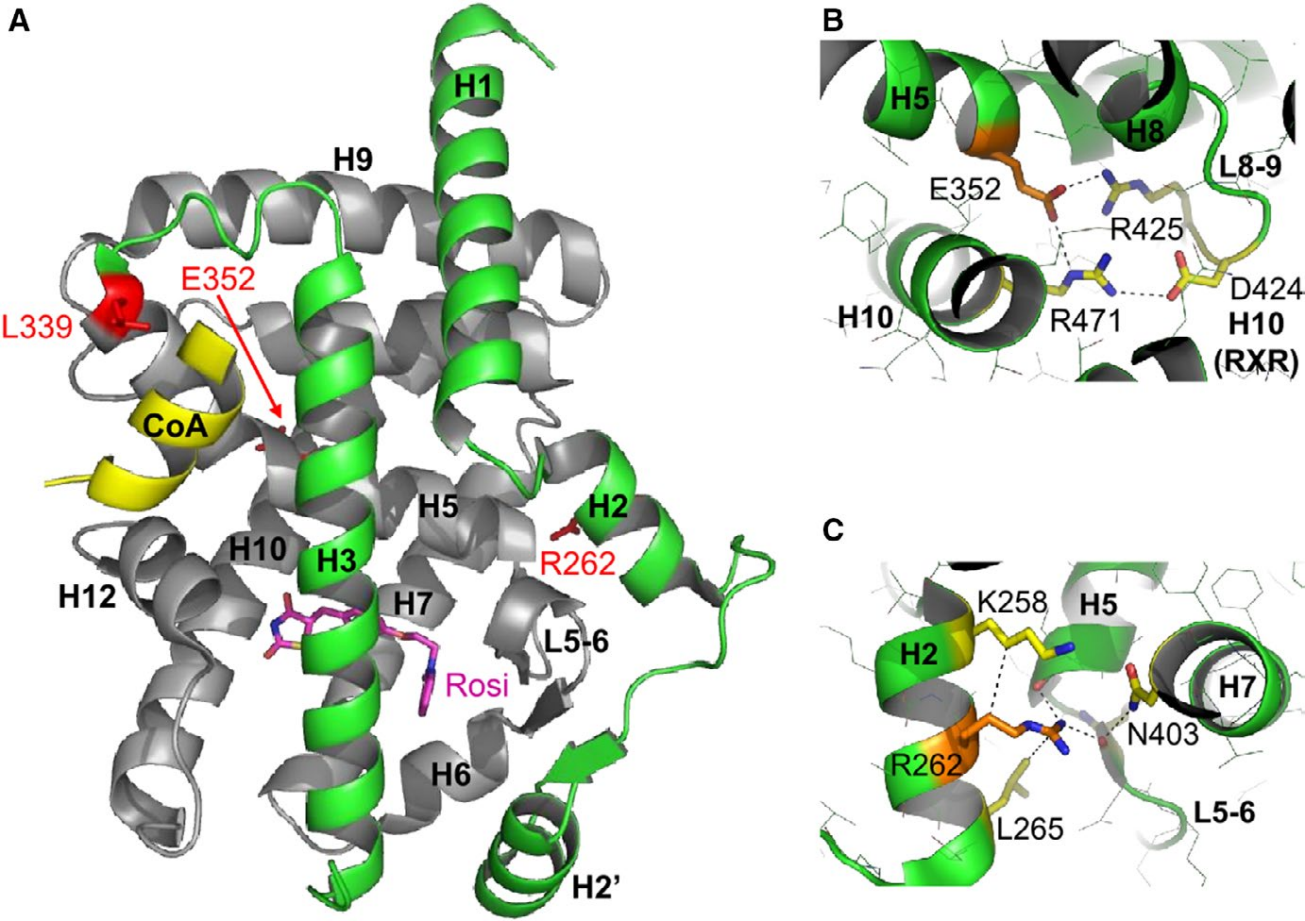
Structural analysis of three PPARG‐LBD mutations. A, L339X, B, E352Q and C, R262G. Overall structure of the LBD of PPARG bound to rosiglitazone (Rosi in magenta) and a short peptide derived from the transcriptional coactivator SRC‐1 (CoA, yellow helix). α‐helices are labelled from H1 at the N‐terminus to H12 at the C‐terminus of the domain. The mutated residues (R262, L339, E352) are displayed as red sticks and labelled. The portion shown in grey (H4 to H12) is missing in the L339X mutant

The effects of mutations in PPARG on cell fusion and migration processes have not been widely studied in the literature. We focused our study on three mutations located in the PPARG LBD that presents dimerization defects and/or impaired ligand‐ and cofactor binding (Figure 5). Here, we first investigated the effect of the E352Q mutation that was initially reported from a case of FPLD3 that resulted in foetal death.^19^ Using the NIH/3T3 cell line, which does not express endogenous PPARG, our results showed that this mutation significantly reduced PPARG activity; interestingly, however, this could be almost fully restored in vitro by adding GW1929, a potent PPARG agonist. This pattern of activity has not been previously reported for other PPARG mutations implicated in FPLD3, such as the L451P mutation (accompanied by general defects in ligand‐mediated cofactor interactions and impaired DNA binding due to reduced RXRα heterodimerization^17^) or the D424N mutation for which a partial restoration in PPARG activity was described following treatment with rosiglitazone, another PPARG agonist.^29^ E352 is located in helix H5, which is one of the structural elements constitutive of the PPARG ligand‐binding pocket (LBP, Figure 5A). E352 is involved in a network of hydrogen bonds and salt bridges with R471 from helix H10 as well as D424 and R425 from loop L8‐9 which are part of the dimerization surface with RXR (Figure 5B). The replacement of a negatively charged glutamic acid by a glutamine in a positively charged environment is likely to perturb the local molecular organization and alter the ligand‐binding and heterodimerization functions of PPARG. Interestingly, treatment with 1 µmol/L of GW1929 appeared sufficient to overcome the effect of the mutation and partially restore PPARG activity (Figure 2D). Another effect of the E352Q mutation studied here was a reduction in VCT fusion into ST, which could be evidence of the involvement of PPARG in the human placental abnormalities that were described by Castell et al in 2012.^19^

Next, we investigated the effect of two other mutations in the LBD of PPARG (R262G and L339X) on cell migration. As shown in Figure 5A, the structural elements supporting the ligand‐binding, coregulator‐binding and dimerization functions are lost in the L339X mutant, rendering the LBD of this PPARG variant non‐functional. Although not directly in contact with the ligand, the R262G mutation (in helix H2) is involved in a network of van der Waals contacts and hydrogen bonds stabilizing the end of H5 and the loop linking H5 and H6 (L5‐6), which both contain residues of the LBP (Figure 5C). By destabilizing the helical conformation of H2 (the glycine amino acid is known to be an α‐helix breaker) and suppressing the interaction network nucleated by R262 (the glycine residue is lacking a side chain), the R262G mutation is very likely to disturb ligand binding and, in turn, receptor activation by endogenous or synthetic ligands. Skin fibroblasts from FPLD3 patients (PPARG mutant) showed reduced migration compared to wild‐type controls. These results could be explained by the different shapes observed between the wild‐type and PPARG‐mutated fibroblasts (fusiform vs flattened, respectively). In general, flattened fibroblasts are non‐polarized/stationary whereas polarized/migrating fibroblasts are fusiform in shape and exhibit lamellipodia or filopodia. Furthermore, vinculin staining revealed a clear increase in focal adhesions in PPARG‐mutated fibroblasts compared to wild‐type. Vinculin is an adaptor protein, which localizes to integrin‐mediated cell‐matrix adhesions, and it has been described as a suppressor of cellular migration,^30^ which is consistent with our results.

In the literature, a dialogue between villous cytotrophoblasts and mesenchymal cells has been described in placenta development,^31^, ^32^ further experiments such as co‐cultures, organoids would be of interests to determine how PPARG‐mutated mesenchymal cells could interfere in VCT fusion: through indirect paracrine signalling (exosomes, EVs, growth factors)? through direct physical interactions via the extracellular matrix? through direct cell‐to‐cell contact?

In conclusion, the evidence presented here clearly shows that a single missense or nonsense mutation in the LBD of PPARG significantly inhibits cell fusion and migration processes.

## CONFLICT OF INTEREST

The authors report no conflict of interest.

## **AUTHOR** **CONTRIBUTIONS**

**Hussein Shoaito:** Investigation (equal); Writing‐original draft (equal). **Sabine Chauveau:** Formal analysis (supporting); Investigation (supporting). **Camille Gosseaume:** Formal analysis (supporting); Investigation (supporting). **William Bourguet:** Formal analysis (supporting); Investigation (supporting). **Corinne Vigouroux:** Resources (supporting); Writing‐review & editing (supporting). **Camille Vatier:** Resources (supporting); Writing‐review & editing (supporting). **Catherine Pienkowski:** Resources (supporting); Writing‐review & editing (supporting). **T. Fournier:** Funding acquisition (equal); Project administration (equal); Resources (equal); Writing‐review & editing (equal). **Severine DEGRELLE:** Conceptualization (lead); Formal analysis (lead); Investigation (lead); Methodology (lead); Project administration (equal); Supervision (lead); Validation (lead); Writing‐original draft (lead); Writing‐review & editing (lead).

## Data Availability

The data that support the findings of this study are available from the corresponding author upon reasonable request.
